# Association between accelerometry measured patterns of sedentary behaviors and functional status in older adults

**DOI:** 10.1007/s40520-023-02644-z

**Published:** 2024-01-28

**Authors:** Jung Yoen Son, Weijiao Zhou, Katelyn E. Webster-Dekker, Deanna J. Marriott, Janet L. Larson

**Affiliations:** 1 School of Nursing, University of Michigan, 400 North Ingalls, Ann Arbor, MI 48109-5482 USA; 2 School of Nursing, Peking University, Beijing, China; 3 School of Nursing, Indiana University, Indianapolis, IN USA

**Keywords:** Sedentary behavior, Sedentary behavior pattern, Sedentary behavior fragmentation, Older adults, Function

## Abstract

**Background:**

Older adults are highly sedentary, and too much sedentary behavior (SB) is associated with negative health effects, but little is known about SB patterns and their associations with functional status.

**Aims:**

To examine the association between objectively measured sedentary behavior time (SBT) and sedentary behavior fragmentation (SBF) and functional status in older adults using the National Health Aging Trends Study (NHATS) dataset, a nationally representative sample from 2021.

**Methods:**

Data from NHATS were analyzed using weighted linear regressions to examine the association between objective measures of SBT (mean hours spent in SB/day during waking hours) and SBF, and six functional variables (difficulties with activities of daily living [ADL], short physical performance battery, hand grip strength, immediate word recall, delayed word recall, and mental health), accounting for sociodemographic, body mass index, and the number of chronic conditions.

**Results:**

A total of 738 individuals from the NHATS were included. Higher SBT was associated with greater difficulties with ADL, poorer short physical performance battery and hand grip strength, lower scores in both immediate and delayed word recall, and poorer mental health. Higher SBF was associated with fewer difficulties with ADL, better short physical performance battery and hand grip strength, a higher score in immediate word recall, and better mental health.

**Discussionand conclusions:**

Greater fragmentation of SB was associated with better function, and increasing SBF may be a useful strategy for mitigating the effects of SB in older adults, but prospective research is needed to support this approach.

**Supplementary Information:**

The online version contains supplementary material available at 10.1007/s40520-023-02644-z.

## Background

High sedentary behavior (SB) in older adults is associated with adverse health effects [1]. Too much SB is associated with poorer cognitive and physical function [1], a higher risk of disability in activities of daily living, instrumental activities of daily living [2], physical frailty [3], and premature mortality [4]. Sedentary behavior is any waking behavior in a sitting or lying position that requires low energy expenditure ($\le$ 1.5 metabolic equivalents) [5]. Evidence indicates older adults are more sedentary than other age groups, [6] spending 9.4 h/day during their waking hours in SB [7]. Given the rapidly aging population worldwide [8], high SB among older adults poses a growing public health concern in many countries.

Previous studies linking SB with health outcomes in older adults mainly focused on the total sedentary time [7, 9], and there is limited research on patterns of SB accumulation, such as sedentary behavior fragmentation (SBF). Sedentary behavior fragmentation is a relatively new concept that quantifies the likelihood of breaking up bouts of sedentary time by transitioning to any activity, exceeding low energy expenditure (> 1.5 metabolic equivalents) [10]. Sedentary behavior fragmentation is distinct from the total volume of SB. For example, one individual may accumulate sedentary time through a few bouts of prolonged sitting, whereas another may accumulate sedentary time in numerous short sitting bouts throughout the day. Although both individuals spend similar amounts of total sedentary time, the number of transitions from sitting to the active state can differ significantly, potentially influencing mental health, and physical and cognitive function. Currently, studies have suggested that higher SBF is associated with a better cardiometabolic health [11, 12]. However, SBF still remains unexplored in association with health and physical and cognitive functional status in older adults.

Reducing SB could be an important target to promote mental health, and physical and cognitive function in older adults. There are no U.S. guidelines for sedentary behavior for older adults, but Canadian guidelines recommend limiting sedentary time to 8 h or less a day [13]. Achieving the recommended levels of total sedentary time may not be realistic for highly sedentary older adults. However, interventions focused on components of SB, such as breaking up bouts of sedentary time and increasing SBF, may be more feasible and effective for older adults because intervention strategies could include simply standing up for brief intervals. Understanding sedentary patterns and their effects on function in older adults may have implications for the development of interventions to target SB.

The aims of this study were to describe the SB pattern among older adults and examine the association between daily mean SBT and SBF and functional variables using the National Health and Aging Trends Study (NHATS) dataset gathered from a nationally representative sample of US older adults.

## Methods

### Data sources and study population

This is a cross-sectional secondary analysis using a dataset from the National Health and Aging Trends Study (NHATS). The NHATS researchers collect data annually from a nationally representative panel of older Medicare beneficiaries (aged $\ge$ 65) living in the community, residential care, and nursing homes in the United States [14]. Data have been collected since 2011. We analyzed the public dataset in Round 11 of the NHATS, which was collected in 2021. In Round 11, the NHATS researchers began collecting physical activity data using wrist accelerometry. The accelerometry sample was selected proportional to the Round 9 analytic weight [15]. Among NHATS participants who were eligible to wear an accelerometer, 872 respondents completed NHATS in Round 11, and 747 returned the accelerometer with usable data [15]. Respondents with at least 3 valid days of accelerometry data were included; nine were excluded, leaving a study sample of 738 participants.

### Measurements

*Functional Variables* Functional variables included difficulty with activities of daily living, lower extremity function, limb muscle strength, cognitive function, and mental health. The difficulty with activities of daily living (ADL) was based on self-reported difficulty with six activities: eating, showering or bathing, using the toilet, getting dressed, getting out of bed, and getting around inside the home [16]. The possible number of difficulties with ADL ranged from 0 to 6, where a higher number indicates a greater disability. Lower extremity function was based on the short physical performance battery (SPPB) tests of gait speed, chair stand, and balance [17]. Performance of each activity scored from 0 to 4, and the total possible score ranged from 0–12, where higher scores indicate a better lower extremity function [17]. Limb muscle strength was based on hand grip strength (in kg) in the self-reported dominant hand. Two measures were taken, and the highest score was used [18]. Cognitive function was based on the immediate word recall score and delayed word recall score. Potential scores for each immediate and delayed word recall ranged from 0 to 10, where a higher score indicates a better cognitive function [19]. Mental health was assessed by summing scores of two items assessing depression from Patient Health Questionnaire-2 and two items assessing anxiety from the Generalized Anxiety Disorder-2 scale [20]. Possible scores ranged from 4 to 16, with a higher score indicating poorer mental health [20]. The reliability and validity of this scale have been supported [21].

*Sedentary behavior* Sedentary behavior was measured with the Actigraph CentrePoint Insight Watch (“Activity Watch”). The NHATS participants were instructed to wear the Activity Watch 24 h a day on self-reported non-dominant wrist for seven consecutive days, except for swimming or bathing lasting longer than 30 min. The accelerometer records wrist movement in units of gravity (g) at a sampling rate of 64 Hz [15]. After the data collection period, participants returned the monitor to a research center via prepaid padded envelopes. The accelerometry data were processed using minute-level epochs by the Johns Hopkins research team [15]. Nonwear time was detected and removed using a 90 consecutive minutes threshold [22]. A valid day was defined as wearing the device $>$ 90% of each day (1296 min a day) [15].

We processed the NHATS accelerometry data (accelerometry detailed file) using the R ARCTOOLS package [23] to obtain SB variables for data collected between 5:01 am and 10:59 pm, an approximation of waking hours [24]. Sedentary behavior was defined as the time spent below a threshold of 1853 counts a minute [25]. A sedentary bout was defined as consecutive minutes in the sedentary state lasting at least 1 min [26]. Two SB variables were used from the NHATS accelerometry dataset: daily mean sedentary time (SBT) and sedentary behavior fragmentation (SBF). The SBF reflected the probability of transitioning from a sedentary state to an active state and was calculated as the reciprocal of the average sedentary bout duration for each study participant (= 1/ mean sedentary bout length) [26]. A higher value indicates a more fragmented SB.

*Covariates* Covariates included body mass index (BMI), the number of comorbidities, and sociodemographic characteristics: age (categorized into 2 age intervals [65–79 and 80 and over]), sex, race/ethnicity (non-Hispanic White and Other), education (less than a college degree and college degree), and marital status (married/partnered and not married), and residence (community and residential care facilities/nursing homes). Body mass index was calculated from participant-reported height and weight. The number of chronic conditions was based on self-reported chronic conditions, including heart attack, heart disease, blood pressure, arthritis, osteoporosis, diabetes, lung, stroke, dementia, and cancer.

### Statistical analysis

Descriptive statistics (the count and percentages for categorical variables, mean and standard deviations for continuous variables) were calculated to summarize covariates (socio-demographics, BMI, the number of comorbidities), six functional variables of interest, and SB variables.

We conducted 12 weighted linear regressions to investigate the relationship between each SB variable and each of 6 functional variables of interest, controlling for 6 sociodemographic characteristics (age, sex, race/ethnicity, educational level, marital status, and residence), the number of comorbidities, and BMI. Each functional variable was considered in its own model. Due to multicollinearity between SB variables (SBT and SBF), we did not include both SB variables in the same model. The complex sample design and sampling weights in Round 11 were accounted for in all analyses of this study. The adjusted coefficients, standard errors, and 95% confidence intervals were computed. P-values < 0.05 were considered significant, but we report p-values to 3 decimal places. All statistical analyses were performed using Stata/BE 17.0 software.

## Results

### Characteristics of the study participants

Table 1 shows the characteristics of the participants. More than half of the participants (60.3%) were younger than 80 years old. Most were community-dwelling (94.3%) with a mean BMI of 28.2 (SD 6.9) kg/m^2^ and a mean of 2.9 (SD 1.3) chronic conditions. The most prevalent chronic condition was high blood pressure, affecting 74.4% of study participants, followed by arthritis (72.6%) and osteoporosis (36.3%). Participants spent an average of 12.7 h/day (SD 2.0) in SB during waking hours (5:01 am-10:59 pm) and had a sedentary fragmentation of 0.11 (SD 0.04). Total active time/day, mean sedentary bout duration/day, and sedentary behavior fragmentation are shown in Table 1.

**Table 1:** Characteristics of participants (mean or proportion)

Characteristics	Total (n = 738)
Socio-demographic characteristics	
Age, n (%)	
Young older (aged ≤ 79)	445 (60.3)
Oldest older (aged ≥ 80)	293 (39.7)
Sex, n (%)	
Female	400 (54.2)
Male	338 (45.8)
Race/Ethnicity, n (%)	
Non-Hispanic White	593 (82.3)
Other	128 (17.8)
Education stats, n (%)	
Less than college degree	466 (65.5)
College degree	256 (35.5)
Marital Status, n (%)	
Not married	370 (50.1)
Married	368 (49.9)
Residence, n (%)	
Living in community	696 (94.3)
Living in care facilities	42 (5.7)
BMI, mean (SD)	28.2 (6.9)
No. of chronic conditions, mean (SD)	2.9 (1.3)
Outcomes of interest	
Physical functions	
No. of difficulty with ADL, mean (SD)	0.8 (1.4)
SPPB, mean (SD)	8.6 (3.4)
Hand grip strength, mean (SD)	27.6 (12.5)
Cognitive functions	
Immediate word recall, mean (SD)	5.2 (1.7)
Delayed word recall, mean (SD)	4.0 (2.1)
Mental Health	
Mental health, mean (SD)	5.6 (2.2)
SB variables	
Daily mean sedentary time, hour/day	12.7 (2.0)
Sedentary fragmentation	0.11 (0.04)
Daily mean sedentary bouts duration, min/day	10.7 (8.4)
Daily mean active time, hour/day	5.3 (2.0)

*SD* standard deviation, *SPPB* short physical performance battery, *ADL* activities of daily living, *SB* sedentary behavior

### Sedentary behavior and functional variables

Table 2 shows the relationships between mean SBT and functional variables. In the weighted adjusted models, higher SBT was significantly associated with greater difficulty with ADL ($\beta$= 0.17, 95% CI [0.10, 0.25], p < 0.001), lower SPPB scores ($\beta$=-0.43, 95% CI [– 0.57, – 0.28], p < 0.001), lower hand grip scores ($\beta$ = – 0.98, 95% CI [– 1.48, – 0.48], p < 0.001), lower immediate word recall scores ($\beta$ = – 0.09, 95% CI [– 0.16, – 0.02], p = 0.013) and delayed word recall score ($\beta$ = – 0.10, 95% CI [-0.19, – 0.01], p = 0.025), and higher mental health scores ($\beta$ = 0.15, 95% CI [0.04, 0.26], p  =  0.007) (see Table 2).

**Table 2:** Weighted estimates of coefficients in linear regression models of sedentary behavior time and functions

	No. of difficulty with ADL $\beta$ (SE)	SPPB $\beta$ (SE)	Hand grip strength $\beta$ (SE)	Immediate word recall $\beta$ (SE)	Delayed word recall $\beta$ (SE)	Mental health score $\beta$ (SE)
SBT (hour)	0.17 (0.04)***	– 0.43 (0.07)***	– 0.98 (0.25)***	– 0.09 (0.04)*	– 0.10 (0.04)*	0.15 (0.05)**
Age						
65–79	Ref	Ref	Ref	Ref	Ref	Ref
80 and older	0.08 (0.13)	– 1.48 (0.28)***	– 4.33 (0.89)***	– 0.83 (0.16)***	– 1.09 (0.18)***	– 0.24 (0.19)
Sex						
Male	Ref	Ref	Ref	Ref	Ref	Ref
Female	0.05 (0.14)	– 0.35 (0.28)	– 14.29 (0.94)***	0.58 (0.12)***	0.69 (0.15)***	0.33 (0.20)
Race/Ethnicity						
Other	Ref	Ref	Ref	Ref	Ref	Ref
White, Non-Hispanic	– 0.49 (0.19)*	1.20 (0.39)**	3.38 (1.16)**	0.44 (0.14)**	0.49 (0.18)**	– 0.11 (0.24)
Education						
No college degree	Ref	Ref	Ref	Ref	Ref	Ref
College degree	– 0.23 (0.12)	0.43 (0.26)	1.13 (1.06)	0.65 (0.13)***	0.73 (0.16)**	– 0.16 (0.20)
Marital Status						
Not married	Ref	Ref	Ref	Ref	Ref	Ref
Married	0.11 (0.13)	0.64 (0.27)*	0.28 (0.81)	– 0.08 (0.12)	0..06 (0.16)	– 0.12 (0.18)
Residence						
Living in care facilities	Ref	Ref	Ref	Ref	Ref	Ref
Living in community	– 0.06 (0.26)	1.40 (0.71)	0.46 (1.74)	– 0.04 (0.25)	0.17 (0.39)	0.23 (0.43)
BMI	0.01 (0.01)	– 0.04 (0.03)	0.15 (0.07)*	0.003 (0.01)	0.00 (0.02)	0.02 (0.02)
No. of chronic conditions	0.21 (0.05)***	– 0.38 (0.10)***	– 0.52 (0.31)	– 0.21 (0.05)***	– 0.24 (0.06)***	0.36 (0.07)***

Models are adjusted for complex survey design and covariates (age, sex, race/ethnicity, education, marital status, residence, BMI, and no. of chronic conditions)

*SBT* sedentary behavior time, SPPB short physical performance battery, *ADL* activities of daily living, *BMI* body mass index, *SE* standard error

*P < 0.05

**P < 0.01

***P < 0.001

Table 3 shows the result of the linear regression models that examine the relationships between SBF and functional variables. In the weighted adjusted models, higher SBF was significantly associated with fewer difficulties with ADL ($\beta$ = – 7.04, 95% CI [-10.54, -3.53], p < 0.001), higher SPPB ($\beta$ = 16.54, 95% CI [9.12, 23.96], p < 0.001), higher hand grip strength ($\beta$ = 38.11, 95% CI [12.26, 63.95], p  =  0.005), higher immediate word recall ($\beta$ = 4.17, 95% CI [0.83, 7.51], p  =  0.015), and lower mental health scores ($\beta$ = – 6.90, 95% CI [-12.19, -1.61], p  =  0.011) (see Table 3). However, SBF was not associated with delayed word recall ($\beta$ = 1.77, 95% CI [– 3.00, 6.54], p  =  0.467).

**Table 3:** Weighted estimates of coefficients in linear regression models of sedentary behavior fragmentation and functions

	No. of difficulty with ADL $\beta$ (SE)	SPPB $\beta$ (SE)	Hand grip strength $\beta$ (SE)	Immediate word recall $\beta$ (SE)	Delayed word recall $\beta$ (SE)	Mental health score $\beta$ (SE)
SBF	– 7.04 (1.75)***	16.54 (3.70)***	38.11 (12.90)**	4.17 (1.67)*	1.77 (2.38)	– 6.90 (2.64)*
Age						
65–79	Ref	Ref	Ref	Ref	Ref	Ref
80 and older	0.14 (0.13)	– 1.58 (0.27)***	– 4.79 (0.94)***	– 0.85 (0.16)***	– 1.10 (0.19)***	– 0.19 (0.20)
Sex						
Male	Ref	Ref	Ref	Ref	Ref	Ref
Female	– 0.06 (0.15)	– 0.13 (– 0.24)	– 13.87 (0.96)***	0.63 (0.12)***	0.75 (0.14)***	0.24 (0.20)
Race/Ethnicity						
Other	Ref	Ref	Ref	Ref	Ref	Ref
White, Non-Hispanic	– 0.44 (0.20)*	1.11 (0.40)*	3.26 (1.18)**	0.42 (0.15)**	0.44 (0.18)*	– 0.07 (0.26)
Education						
No college degree	Ref	Ref	Ref	Ref	Ref	Ref
College degree	– 0.25 (0.13)	0.46 (0.28)	1.23 (1.07)	0.65 (0.13)***	0.76 (0.17)***	– 0.15 (0.21)
Marital Status						
Not married	Ref	Ref	Ref	Ref	Ref	Ref
Married	0.07 (0.13)	0.77 (0.29)*	0.43 (0.83)	– 0.07 (0.12)	0.11 (0.16)	– 0.15 (0.17)
Residence						
Living in care facilities	Ref	Ref	Ref	Ref	Ref	Ref
Living in community	– 0.11 (0.26)	1.60 (0.72)*	0.57 (1.80)	– 0.02 (0.26)	0.12 (0.40)	0.18 (0.45)
BMI	0.02 (0.01)	– 0.06 (0.03)*	0.12 (0.07)	0.0005 (0.01)	0.002 (0.02)	0.03 (0.02)
No. of chronic conditions	0.24 (0.06)***	– 0.49 (0.11)***	– 0.53 (0.35)	– 0.22 (0.05)***	– 0.28 (0.07)***	0.37 (0.09)***

Models are adjusted for complex survey design and covariates (age, sex, race/ethnicity, education, marital status, residence, BMI, and no. of chronic conditions)

*SBF* sedentary behavior fragmentation (sedentary to active transition probability), *SPPB* short physical performance battery, *ADL* activities of daily living, *BMI* body mass index, *SE* standard error

*P < 0.05

**P < 0.01

***P < 0.001

## Discussion

In this study, lower SBT and higher SBF were associated with fewer difficulties with ADL, better physical and cognitive function, and better mental health. To our knowledge, this is the first study to explore the associations between SBF and functional variables in older adults from a nationally representative US sample.

This study supports the existing evidence that US older adults spend most of their waking hours in SB. The SBT in the present study is consistent with a previous study using the same accelerometer [24] but higher than the average sedentary time reported in a systemic review of community-dwelling older adults (SBT  =  9.4 h) [7]. The inconsistent findings may be explained by the heterogeneity of measurement tools. For example, the review was primarily based on studies with waist-worn ActiGraph, whereas the NHATS used wrist-worn ActiGraph, in which processing data reduction is less established [27, 28].

It is well-established that low sedentary time is protective for health, physical function, and depression [29–35]. We confirmed the association between low SBT and better physical and cognitive function and mental health. The strength of the association between SBT and SPPB in this study indicates a potentially clinically meaningful effect. For example, for every 1-h decrease in SBT, we observed an increase of 0.43 points in SPPB scores. The result is within the ranges of minimally significant changes in 0.3–0.8 points for the SPPB [36].

The observed positive relationship between SBF and function is consistent with prior research focusing on breaks in SB. A greater number of SB breaks was associated with lower cardiometabolic risks [37], better physical performance [38], and a lower likelihood of disability in instrumental activities of daily living [2]. The number of SB breaks captures the number of interruptions in SB but doesn’t reflect the duration of sitting bouts. However, SBF captures the tendency to stay in SB by capturing sedentary breaks and the duration of sitting bouts [26]. This supported the need for tailored interventions addressing SBF to improve the functions of sedentary older adults.

The inconsistent relationship between SBT and SBF and cognitive variables in this study is consistent with prior reports. For instance, a systemic review of 18 studies demonstrated varied and inconclusive evidence for the association between SB and cognitive function in older adults [39]. Similarly, another study reported that sedentary time had a minor association with executive functions, but prolonged sedentary time was not associated with any cognitive test scores [40]. The inconsistent results may be attributed to the effects of different types of SB on cognition in older adults [41]. Mentally active SB (e.g., reading or writing) may be protective for cognition, contrary to the harmful effects of mentally passive SB (e.g., watching TV) on cognition [41].

The above findings highlight the potential benefits of both reducing total sedentary time and increasing SBF. Prolonged sitting hours with frequent SB breaks may be targeted through interventions, especially in highly sedentary older populations or potentially individuals who have mild mobility issues in community or care settings. Clinicians may encourage older adults to reduce sedentary time, especially prolonged sedentary time, and suggest more transitions from sitting to standing. This may provide an opportunity to curb SB for sedentary older adults.

This research advances the science of SB in older adults, and it has several strengths. We used a national dataset collected from a nationally representative sample of US older adults. The use of objectively measured SB reduced the risk of recall bias and is more accurate than self-reported measures. Limitations of this research include a cross-sectional analysis that cannot establish the causality of the relationships between SB patterns and functional variables. Our data did not include information about individual sleep information, affecting the estimates of the SB variables.

## Conclusion

This is the first to identify the associations between SBF and functional status in older adults from a nationally representative US sample. Older individuals with higher sedentary time and lower sedentary fragmentation tend to report greater difficulties with ADL, poorer upper and lower extremity function, poorer cognitive function, and worse mental health. Our study suggests that interventions may focus on reducing overall sedentary time and increasing sedentary behavior fragmentation to promote functions in older adults, but prospective research is needed to confirm this conclusion.

### Supplementary Information

Below is the link to the electronic supplementary material.

Supplementary file1 (DOCX 71 KB)

## Data Availability

Publicly available data from the National Health and Aging Trends Study (NHATS) were analyzed for this research. www.nhats.org
