# Supplementary material for: Association between accelerometry measured patterns of sedentary behaviors and functional status in older adults
Source: Aging Clin Exp Res. 2024 Jan 28;36(1):11. doi: 10.1007/s40520-023-02644-z (PMC10822805; doi:10.1007/s40520-023-02644-z)
Supplement: Supplementary file 1 — Supplementary file1 (DOCX 71 KB) [file 40520_2023_2644_MOESM1_ESM.docx]

Supplemental Table 1. Measurement table

|  | **Variables** | **Measurements** |
| --- | --- | --- |
| Independent variables | Sedentary behavior time (SBT) | Accelerometry measured mean hours spent being inactive a day during waking hours |
|  | Sedentary behavior fragmentation (SBF) | Accelerometry measured the probability of transitioning from a sedentary to an active state during waking hours |
| Outcome of interest | The number of difficulties in activities of daily living (ADL) | The number of self-reported difficulty with the following validated six items: eating, showering or bathing, using the toilet, getting dressed, getting out of bed, and getting around inside the home |
|  | Lower extremity function | Short physical performance battery (SPPB) score |
|  | Limb muscle strength | Hand grip strength score |
|  | Cognitive function | Immediate word recall score |
|  |  | Delayed word recall score |
|  | Mental health | Patient Health Questionnaire-2 and Generalized Anxiety Disorder-2 scale score |
